# Fibrinolysis for Apical Thrombus as a Bridge to Emergent Valve-in-Valve Transcatheter Aortic Valve Replacement

**DOI:** 10.1016/j.jaccas.2025.106066

**Published:** 2025-11-21

**Authors:** Nicholas J. Valle, Omar A. Saleh, Mani C. Akunuri, Parth K. Parikh, Joshua A. Cohen, Deepak R. Talreja, Matthew R. Summers

**Affiliations:** a Macon and Joan Brock Virginia Health Sciences at Old Dominion University, Eastern Viginia Medical School, Internal Medicine Residency Program, Norfolk, Virginia, USA; b Macon and Joan Brock Virginia Health Sciences at Old Dominion University, Eastern Viginia Medical School, Norfolk, Virginia, USA; c Department of Cardiology, Sentara Medical Group, Sentara Health, Virginia Beach, Virginia, USA

**Keywords:** acute valve syndrome, aortic valve, cardiogenic shock, fibrinolysis, intracardiac echocardiography, TAVR, thrombus, valve-in-valve

## Abstract

**Background:**

Left ventricular (LV) thrombus is traditionally a contraindication to transcatheter aortic valve replacement due to embolic risk. Consequently, management of valvular cardiogenic shock complicated by LV thrombus identifies unique challenges that require innovative strategies.

**Case Summary:**

A 58-year-old woman with critical bioprosthetic aortic stenosis, cardiogenic shock, and large LV thrombus (2.8 × 2.6 cm) underwent successful valve-in-valve transcatheter aortic valve replacement after systemic fibrinolysis bridge therapy.

**Why Beyond the Guidelines:**

Current guidelines contraindicate transcatheter aortic valve replacement in patients with LV thrombus. This case demonstrates the novel use of low-dose, slow infusion tissue plasminogen activator as a bridge therapy to enable life-saving intervention.

**Discussion:**

Pulsed delivery of small doses of systemic fibrinolytic with hemodynamic and echocardiographic monitoring may be suitable as a bridge to definitive management in valvular shock patients with LV thrombus. Minimizing instrumentation during structural heart procedures complicated by LV thrombus is critical.

**Take-Home Messages:**

Low-dose, slow-infusion fibrinolysis coupled with echocardiographic monitoring may serve as bridge therapy to urgent structural cardiology intervention in carefully selected patients with LV thrombus and cardiogenic shock.

## Graphical Abstract

**Figure:**
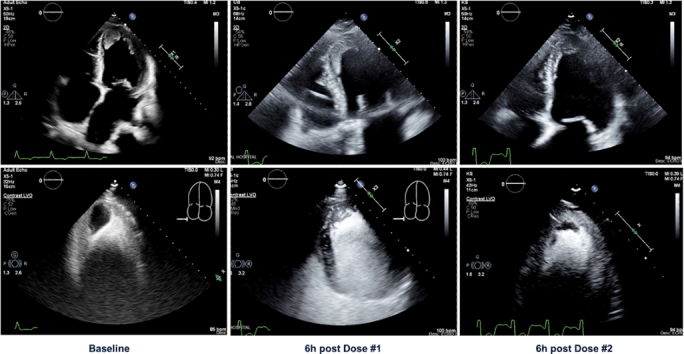

Left ventricular (LV) thrombus contraindicates transcatheter aortic valve replacement (TAVR) due to embolic risk.^1^ However, nonsurgical patients with valvular cardiogenic shock (CS) require urgent valve correction for survival. The presence of LV thrombus complicates management, often prohibiting intervention despite the urgent need in cases of critical bioprosthetic degeneration. We present the novel use of tissue plasminogen activator (tPA) as bridge to valve-in-valve (ViV) TAVR in a patient who presented with critical bioprosthetic stenosis and valvular CS complicated by large LV thrombus.

**Take-Home Messages**

• Pulsed infusions of 25 mg tPA over 6 hours can effectively reduce LV thrombus burden in cardiogenic shock patients requiring urgent structural intervention; however, fibrinogen monitoring should be incorporated.
• Small coronary balloons for true orifice predilation combined with intracardiac echocardiography guidance can facilitate safe valve-in-valve TAVR while minimizing thrombus disruption risk.
• The use of 2 cerebroembolic protection devices to cover all major branches of the aortic arch may benefit patients undergoing high-risk transcatheter heart procedures.

## Case Summary

**Figure 1:**
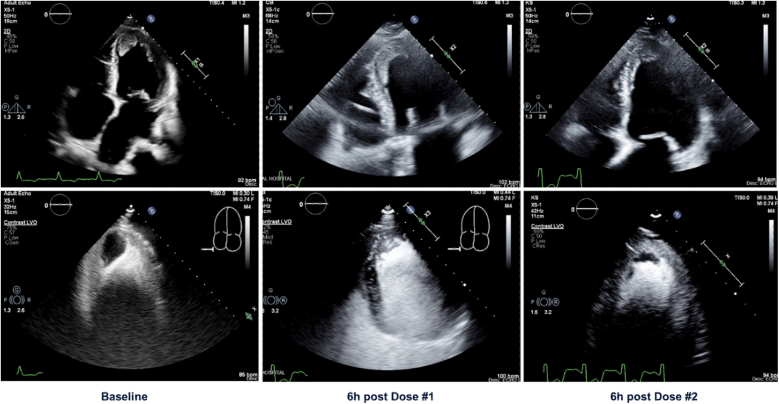
Echocardiographic Projections With and Without Contrast Highlighting Thrombus Size at Baseline (2.8 × 2.6 cm) and After the First (3.4 × 1.02 cm) and Second (2.03 × 0.8 cm) 25 mg tPA Infusions The third dose of tissue plasminogen activator (tPA) was stopped early due to right upper extremity hematoma, and an additional echocardiogram was not obtained.

A 58-year-old woman with a history of aortic regurgitation and ascending aortic aneurysm with combined surgical bioprosthetic aortic valve replacement (21-mm CE Magna Ease; Edwards) and aortic arch repair in 2011 presented to the hospital with progressively worsening dyspnea. Echocardiogram revealed critical aortic stenosis (peak velocity: 4.5 m/s, mean gradient: 51 mm Hg) secondary to bioprosthetic degeneration, newly reduced ejection fraction (15%), and a mobile LV thrombus (2.8 × 2.6 cm, area: 7.28 cm^2^) (Figure 1, Video 1). She subsequently developed valvular CS (cardiac index: 1.6).

Surgical risk was deemed prohibitive. Advanced heart failure therapies and transplant were deferred due to cognitive assessment findings. ViV-TAVR with SENTINEL Cerebral Protection System (Boston Scientific) was then considered. At our center's multidisciplinary heart conference, fibrinolytic debulking of the thrombus as a bridge to intervention was proposed. A protocol from the SAFE-PVT Trial (Surgery Compared to Fibrinolytic Therapy for Symptomatic Left-Sided Prosthetic Heart Valve Thrombosis) was adopted.^2^ Fibrinolytic agent (tPA), dosage, duration of infusion, and interval between infusions were directly adopted from this trial protocol. The approach was discussed with the patient's surrogate who elected to proceed.

A total of three 6-hour pulsed infusions of 25 mg tPA were attempted. The first dose successfully reduced thrombus dimensions by 52% to an area of 3.468 cm^2^ (3.4 × 1.02 cm) (Figure 1, Video 2) without complication. After echocardiogram had been obtained, a second infusion was administered and was well tolerated. During infusion of the third dose, the patient developed a right upper extremity hematoma, and the infusion was stopped. Our final preprocedural echocardiogram was notable for a further decline in thrombus size to an area of 1.624 cm^2^ (2.03 × 0.8 cm) (Figure 1, Video 3).

**Figure 2:**
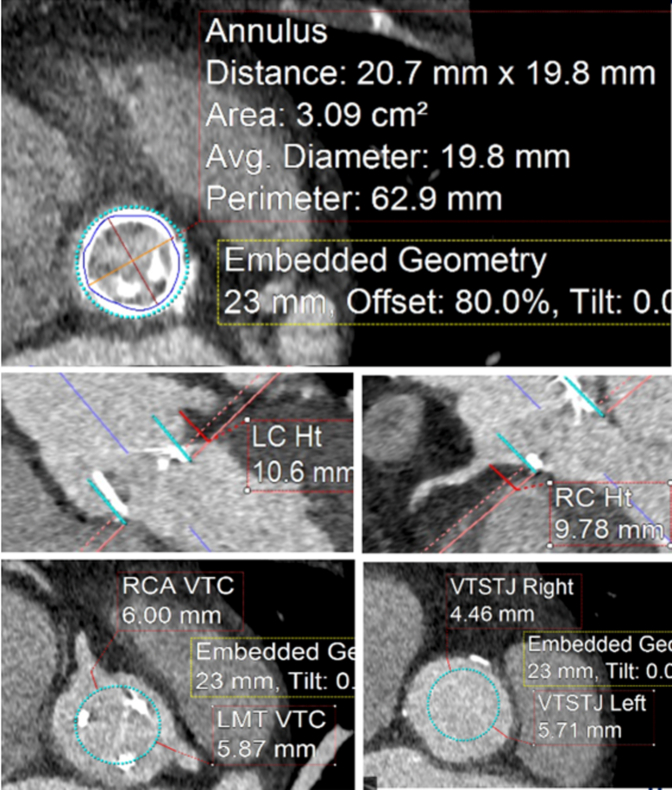
Transcatheter Aortic Valve Replacement Protocol Computed Tomography Angiography Images With Planning Data for Valve-in-Valve Transcatheter Aortic Valve Replacement The RCA height was <10 mm (9.78), indicating increased risk of CO. RCA VTc and VTstj were 0.6.00 mm and 4.46, respectively. Also shown are left main trunk height (10.6 mm), VTc (5.87 mm), and VTstj (5.71 mm). CTA = computed tomography angiography; LC = left coronary; LMT = left main trunk; RC = right coronary; RCA = right coronary artery; VTc = virtual transcatheter heart valve-to-coronary distance; VTstj = virtual transcatheter heart valve-to-sinotubular junction distance.

**Figure 3:**
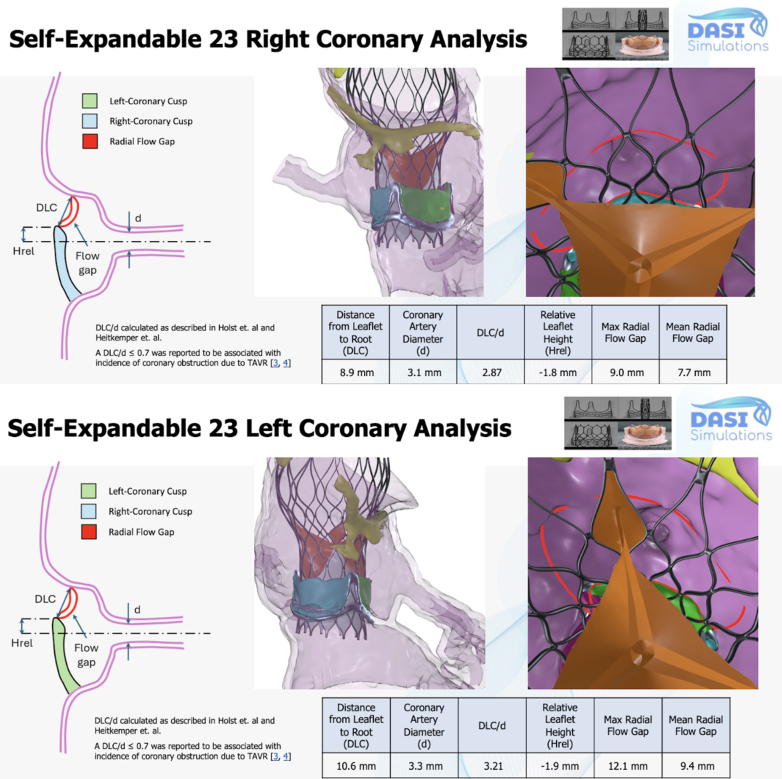
DASI Preprocedural Modeling Analysis Based on the Patient's Pre-Transcatheter Aortic Valve Replacement Computed Tomography Angiography Data The DLC/d ratio is shown for both the right coronary artery (2.87) and left main trunk (3.21). A DLC/d ≤ 0.7 is associated with increased incidence of coronary obstruction.^3^ Balloon-expandable and self-expanding valves of various sizes were tested. A 23-mm self-expanding valve was ultimately chosen because it had the most favorable expected hemodynamics and CO risk profile. Given these findings, leaflet modification was not planned. CO = coronary artery occlusion; CTA = computed tomography angiography; d = coronary artery diameter; DLC = distance from native aortic valve leaflet to coronary ostium; Hrel = relative leaflet height.

**Figure 4:**
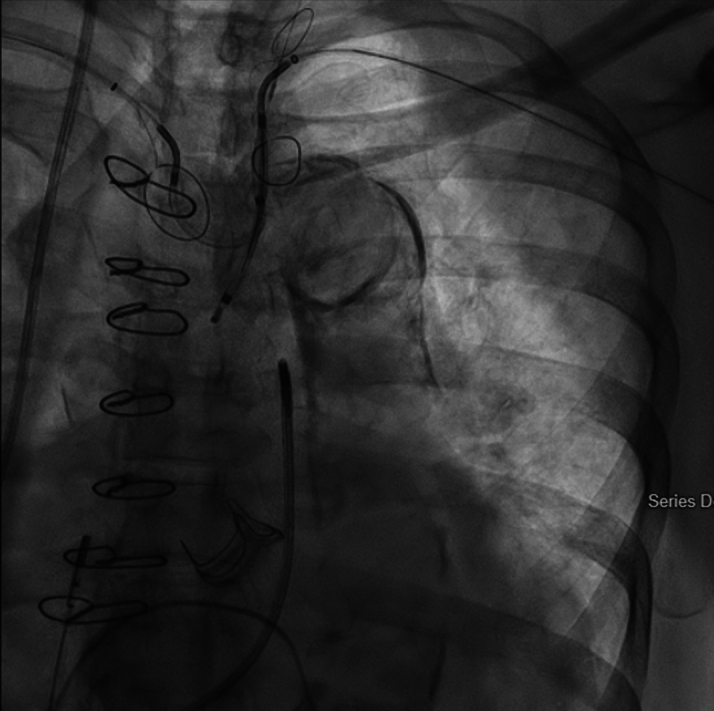
Radiographic Projection Demonstrating the Position of 2 SENTINEL Cerebroembolic Protection Devices With Baskets Deployed in the Brachiocephalic Trunk, Left Common Carotid Artery, and Left Subclavian Artery The right-sided device was introduced via the right ulnar artery and deployed in the usual fashion. The left-sided device was introduced via the left ulnar artery. The proximal basket was then deployed within the left subclavian artery, and the distal basket was left undeployed.

**Figure 5:**
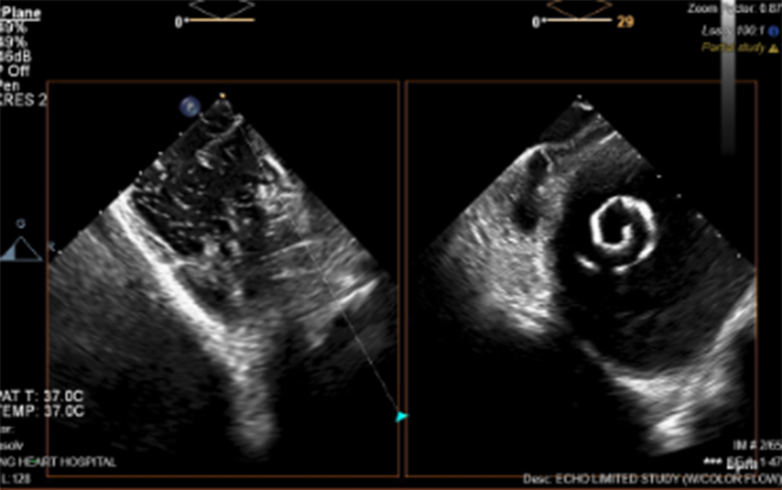
Intracardiac Echocardiography Visualization of Safari Wire Within the LV Cavity Visualization of equipment during this case was imperative to minimize instrumentation of the left ventricular (LV) thrombus.

The patient underwent TAVR protocol computed tomography angiography the year before her acute presentation before being lost to follow-up. Given low right coronary height (9.78 mm), our team leveraged artificial intelligence (AI)–guided preprocedural simulation and computational modeling (DASI Simulations) to improve our assessment of the patient's risk of coronary obstruction and need for leaflet modification (Figures 2 and 3). A 23-mm Medtronic Evolute FX+ was chosen in part due to favorable AI modeling outputs that indicated a low right coronary artery obstruction risk without leaflet modification (Distance from native aortic valve Leaflet Cusp to coronary ostium/coronary artery diameter [DLC/d]_RCA_ = 2.87) (Figure 3).^3^ ViV TAVR was then performed in a hybrid operating room. Bilateral radial arteries were occluded from prior procedures; thus, the bilateral ulnar arteries were accessed for dual SENTINEL device placement. The right-sided device was deployed first in typical fashion. The left-sided device was then deployed via the left ulnar artery. The proximal filter was rested in the left subclavian artery with the distal filter left undeployed (Figure 4). Although the patient's bioprosthetic degeneration limited our catheter advancement across the true orifice, we were ultimately able to traverse the degenerated valve with a Glidewire (Terumo) and predilate the bioprosthesis orifice with a 4.0-mm Charger balloon (Boston Scientific). This technique facilitated subsequent LV access with a multipurpose angiographic catheter that was exchanged for a Safari wire (Boston Scientific). Biplane intracardiac echocardiography (Phillips) was used in conjunction with an right anterior oblique projection to provide a longitudinal view of the LV apex, enhancing our ability to monitor LV wire positioning near the thrombus (Figure 5, Video 4). The transvalvular gradient was measured at 80 mm Hg despite CS.

**Figure 6:**
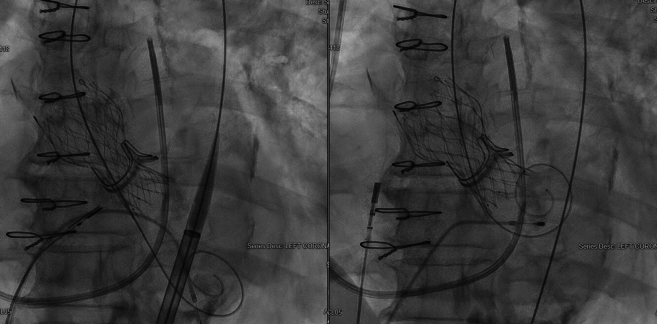
Evolute FX+ After Successful Deployment at 6-mm Depth Initial constraint on the valve frame (left) was significantly improved with postdilation using a 22-mm balloon (right). Initial predilation was avoided to minimize instrumentation; however, postdilation became necessary given valve constrain after deployment.

The valve delivery system was then advanced across the aortic valve. Predilation was deferred to minimize instrumentation. Due to the procedural complexity, we ultimately opted for a deployment depth of 6 mm to reduce the overall procedure time and to minimize the patient's risk of LV thrombus dislodgement. The new valve was significantly constrained after deployment; as a result, we opted for postdilation with a 22-mm balloon (Figure 6). Postdeployment gradients were <10 mm Hg with excellent hemodynamics. Echocardiography revealed trivial paravalvular leak, and our final angiography showed no complications.

## Why Beyond the Guidelines

Current American College of Cardiology/American Heart Association guidelines contraindicate TAVR in patients with LV thrombus, recommending surgery or anticoagulation. There are no guidelines describing use of tPA for LV thrombus reduction as a bridge to TAVR in CS. Our case demonstrates a potential salvage therapy for high-risk patients who otherwise lack treatment options.

## Case Outcome and Follow-Up

After ViV TAVR, the patient had no evidence of neurologic sequelae and was weaned off vasopressor and inotropic support by postprocedural day 3. Transthoracic echocardiography on day 12 showed LV ejection fraction improved to 30% with only trace paravalvular leak; the LV thrombus measured 2.81 cm^2^. The patient was safely discharged home on anticoagulation.

## Discussion

**Figure 7:**
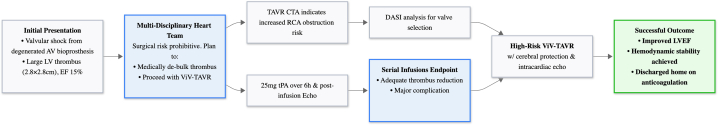
Flowchart Outlining Preprocedural Planning and Medical Decision-Making Administration of serial 6-hour infusions of 25 mg tissue plasminogen activator (tPA) was adopted from the protocol outlined in the SAFE-PVT Trial (Surgery Compared to Fibrinolytic Therapy for Symptomatic Left-Sided Prosthetic Heart Valve Thrombosis).^4^ Serial echocardiograms were used to monitor thrombus response to each round of infusions. Multidisciplinary discussion throughout management was critical. The patient tolerated 2 rounds of tPA infusion. The third transfusion was halted on development of a right upper extremity hematoma. CTA = computed tomography angiography; EF = ejection fraction; LV = left ventricular; LVEF = left ventricular ejection fraction; RCA = right coronary artery; TAVR = transcatheter aortic valve replacement; ViV-TAVR = valve-in-valve transcatheter aortic valve replacement.

The management of valvular CS complicated by LV thrombus presents a therapeutic dilemma. Current American College of Cardiology/American Heart Association guidelines contraindicate TAVR in the presence of LV thrombus due to embolic risk, recommending anticoagulation or surgical intervention.^1^ This case represents the first reported use of systemic fibrinolysis as a bridge to successful ViV TAVR in a patient with critical bioprosthetic stenosis, CS, and a large LV thrombus (Figure 7).^7^

Our patient with severe aortic stenosis (mean gradient: 80 mm Hg), severely reduced LV function (ejection fraction: 10%-15%), large LV thrombus, and CS required emergent valve correction. Our fibrinolysis protocol, adapted from the SAFE-PVT trial,^2^ used pulsed doses of 25 mg tPA administered over 6 hours. This approach differs markedly from conventional tPA protocols for acute stroke or pulmonary embolism, which use larger doses over shorter periods.^4^^,^^5^ Our use of the protocol successfully reduced the patient's thrombus area by 78% (from 7.28 to 1.62 cm^2^), potentially minimizing embolic risk during ViV TAVR. The development of an upper extremity hematoma during the third infusion underscores the importance of monitoring fibrinogen levels, a modification we would recommend in future cases, because levels <220 mg/dL have been associated with elevated risk of severe bleeding.^6^

Previous reports have described successful TAVR in CS with LV thrombus using cerebral protection and intracardiac echocardiography guidance.^7^ However, thrombus size was not mentioned in these cases. Chu et al^8^ reported using tPA for bioprosthetic valve thrombosis with initial success; however, their patient suffered cardiac arrest 48 hours posttreatment. Our case demonstrates that with careful patient selection and close monitoring, fibrinolysis can serve as an effective bridge to definitive intervention.

The technical complexity of this procedure was compounded by the patient's bioprosthetic degeneration, making LV access challenging. We intentionally deferred predilation of the degenerated valve to minimize instrumentation, with the goal of favorable hemodynamics on self-expanding valve deployment. Unfortunately, significant postdeployment valve constraint made postdilation unavoidable. We similarly chose to accept less than optimal valve deployment depth (6 mm) to reduce procedure time and reduce instrumentation near the thrombus. This procedural approach, coupled with intracardiac echocardiography for real-time visualization, minimized thrombus manipulation while achieving acceptable valve deployment position. Our use of 2 SENTINEL devices to provide total protection over the main aortic arch branches is not a widely described technique, and prospective data are needed to validate its utility, especially in procedures with high cardioembolic risk.

The integration of AI-guided preprocedural simulation proved valuable for patient valve selection and coronary obstruction risk assessment. Because ViV TAVR procedures have inherently higher rates of intraprocedural modifications than native TAVR, advanced planning tools become particularly crucial in emergent and complex cases.^9^ The ongoing development of simulation-based valve selection technology may continue to optimize patient periprocedural planning, potentially reducing instrumentation in thrombus-complicated cases as in the case of our patient.

Our patient's recovery, weaning from hemodynamic support by day 3 and with postprocedural LV ejection fraction improvement from 15% to 30%, validates the potential of our approach in patients with limited options. Furthermore, our patient's peak-to-peak gradient of 80 mm Hg despite her severely reduced ejection fraction suggested the significant potential for myocardial recovery after relief of valvular obstruction, which proved accurate.

## Conclusions

This case demonstrates that low-dose, slow-infusion fibrinolysis can successfully bridge carefully selected patients with LV thrombus and CS to urgent ViV TAVR. The pulsed administration of 25 mg tPA over 6 hours, with close echocardiographic and hemodynamic monitoring, achieved substantial thrombus reduction while maintaining an acceptable safety profile. Coupled with our technique to minimize thrombus instrumentation, our approach enabled life-saving intervention in a patient without additional treatment options. As structural heart interventions expand to increasingly complex patients, further studies are needed to establish the safety and efficacy of fibrinolytic protocols for LV thrombus management.

## Funding Support and Author Disclosures

Publication fees were provided by Sentara Health, Virginia Beach, Virginia, USA. Dr Cohen receives consulting and proctoring income from Abbott Pharmaceuticals. Dr Talreja receives speaking, consulting, and proctoring income from Medtronic, Abbott Pharmaceuticals, Abiomed, Bristol Myers Squibb, Novartis, and Amgen. Dr Summers receives speaking, consulting, and proctoring income from Medtronic and Abbott Pharmaceuticals; and serves on the advisory board of Abbot Pharmaceuticals, Medtronic, Picardia, DASI, Shockwave, and Abiomed. All other authors have reported that they have no relationships relevant to the contents of this paper to disclose.

## Appendix

Video 1 Echocardiogram of Initial Left Ventricular Thrombus Burden (2.8 × 2.6 cm)
